# Gegen Qinlian Decoction Attenuates High-Fat Diet-Induced Steatohepatitis in Rats via Gut Microbiota

**DOI:** 10.1155/2018/7370891

**Published:** 2018-12-23

**Authors:** Yi Guo, Pang-hua Ding, Li-juan Liu, Lei Shi, Tang-you Mao, Jun-xiang Li, Yun-liang Wang

**Affiliations:** ^1^Gastroenterology Department, Dongzhimen Hospital, Beijing University of Chinese Medicine, Beijing, 100700, China; ^2^Gastroenterology Department, Dongfang Hospital, Beijing University of Chinese Medicine, Beijing, 100078, China; ^3^Beijing University of Chinese Medicine, Beijing, 100029, China; ^4^Gastroenterology Department of Traditional Chinese Medicine, China-Japan Friendship Hospital, Beijing, 100029, China

## Abstract

Gut microbiota play an important role in modulating energy contribution, metabolism, and inflammation, and disruption of the microbiome population is closely associated with chronic metabolic diseases, such as nonalcoholic fatty liver disease (NAFLD). Gegen Qinlian decoction (GGQLD), a well-known traditional Chinese herbal medicine (CHM), was previously found to regulate lipid metabolism and attenuate inflammation during NAFLD pathogenesis. However, the underlying mechanism of this process, as well as how the gut microbiome is involved, remains largely unknown. In this study, we investigated the effect of varying doses of GGQLD on the total amount and distribution of gut bacteria in rats fed a high-fat diet (HFD) for 8 weeks. Our analysis indicates that Oscillibacter and Ruminococcaceae_g_unclassified are the dominant families in the HFD group. Further, HFD-dependent differences at the phylum, class, and genus levels appear to lead to dysbiosis, characterized by an increase in the Firmicutes/Bacteroidetes ratio and a dramatic increase in the Oscillibacter genus compared to the control group. Treatment with GGQLD, especially the GGQLL dose, improved these HFD-induced changes in intestinal flora, leading to increased levels of Firmicutes, Clostridia, Lactobacillus, bacilli, and Erysipelotrichales that were similar to the controls. Taken together, our data highlight the efficacy of GGQLD in treating NAFLD and support its clinical use as a treatment for NAFLD/NASH patients.

## 1. Introduction

Nonalcoholic fatty liver disease (NAFLD) is a type of liver disease that includes simple hepatic steatosis, nonalcoholic steatohepatitis (NASH), and irreversible cirrhosis [1]. NAFLD affects both children and adults worldwide and its prevalence is rapidly increasing in parallel with the dramatic rise in obesity, diabetes [2, 3], hypertension [4], and dyslipidemia [5]. Indeed, the prevalence of NAFLD was estimated to be 20-30% in Western countries and 5-18% in Asia [6] and was reported to be as high as 80% in patients with obesity while being only 16% in individuals with a normal BMI and no metabolic risk factors [7].

Although NAFLD is a growing challenge worldwide, its pathogenesis is not fully understood and the therapeutic options for patients are limited [8]. Disease pathogenesis was initially explained by the "two-hit" hypothesis, but this theory failed to explain various NAFLD-related molecular changes, resulting in the adoption of the “multiple-hit” hypothesis, which takes into account the complex and multifactorial aspects of the disease [9]. Although a combination of "hits" is likely necessary, each of these risk factors is still ultimately related to the hepatic accumulation of lipids caused by a high-fat diet (HFD) in combination with a sedentary lifestyle [10]. This includes insulin resistance, hormonal changes, and altered genetic factors.

Interestingly, the human gut microbiome, which includes 10–100 trillion microorganisms, has also been shown to play an important role in NAFLD pathogenesis [11]. In fact, a recent study showed that patients with small intestinal bacterial overgrowth (SIBO) have an increased risk of hepatic steatosis [12]. Obesity, which is an independent NAFLD risk factor, has also been associated with gut dysbiosis [13, 14]. Furthermore, the link between the gut microbiota and NASH development was also investigated in mice fed a methionine choline-deficient diet, and the mechanism appears to involve disruption of the NLRP3 or NLRP6 inflammasome which induces colonic inflammation and NASH [15]. SIBO observed in NASH patients is also associated with Toll-like receptor (TLR) 4 expression and the release of interleukin-8 [16].

Although bacteria such as Bacteroidetes and Firmicutes make up the bulk of the gut microflora, nonbacterial organisms such as resident archaeal, fungal, and viral populations might also play a significant function [17]. Moreover, obesity appears to be linked to a decline in microbial diversity in the gut that is accompanied by an increase in the ratio of Firmicutes to Bacteroidetes [18]. Taken together, these and other studies suggest that gut microflora dysbiosis may increase gut permeability and hepatic exposure to injurious substances. These changes could greatly affect NAFLD development and progression as well as the responsiveness of patients to therapeutic strategies.

The proposed NAFLD management strategies often include lifestyle modifications and pharmaceutical interventions [7, 19, 20]. However, compliance with long-term lifestyle modification is poor and most medicines have adverse effects, which limit their usage [21]. Although there are huge expectations regarding the use and effectiveness of probiotics in modulating gut microbiota, the lack of solid evidence precludes their implementation in the management of NAFLD/NASH [22, 23]. Thus, it is necessary to develop novel strategies with fewer side effects and high therapeutic efficiency.

Chinese herbal medicine (CHM) has been used in China and other Asian countries for thousands of years and its use is now spreading worldwide. A unique and basic feature of CHM is the use of multicomponent herbal formulas to ameliorate various abnormalities and diseases. For example, formulas like Linggui Zhugan [24], Yinchenhao [25], and Gegen Qinlian decoction (GGQLD) [26] have been used to treat NAFLD. GGQLD, a well-known traditional CHM from the Treatise on Febrile Diseases, consists of* Kudzu root*,* Rhizoma coptidis, Scutellaria baicalensis Georgi, *and* Glycyrrhizae Radix* and is widely used to clinically treat NASH. This formula has been used for thousands of years in clinic. Besides, we have not discovered specific side effects and contraindications of GGQLD in the process of document retrieval and our experiments. Therefore, GGQLD, which is under conventional dose, has been regarded as a kind of safe and potential treatment in treating NASH until now. In our previous studies, we found that GGQLD has an anti-inflammatory effect [27, 28], which could regulate lipid metabolism disorders and improve liver histology of the rats during NAFLD pathogenesis [26, 29]. In addition, the active components of GGQLD, including baicalin, glabridin, and berberine, have been shown to alleviate inflammation and oxidative stress in vivo and in vitro [30, 31]. Although gut microflora is considered a critical “organ” that participates in nutrient metabolism and immunity in the host [32–34], the effects of GGQLD on gut microflora have not been evaluated.

In the present study, we established a HFD-induced rat model of NASH and administered GGQLD in different dosages to examine its effects and underlying mechanisms, focusing on changes in gut microflora. This involved monitoring the total amount and distribution of bacteria in the gut as well as the relative abundance of different taxa and presence of specific harmful microorganisms in both untreated and GGQLD-treated NASH rats. To our knowledge, this is the first time the effects of GGQLD have been assessed with regard to gut microflora in NAFLD/NASH.

## 2. Materials and Methods

### 2.1. Preparation of GGQLD and Glutamine

GGQLD granules were provided by the Pharmacy Department of Dongfang Hospital, Beijing University of Chinese Medicine (Beijing, China). The granules consisted of the following ingredients:* Kudzu root* (24 g),* Rhizoma coptidis* (9 g),* Scutellaria baicalensis Georgi* (9 g), and* Glycyrrhizae Radix* (6 g). Glutamine (GLU) was purchased from AMRESCO Co., Ltd. (Missouri, TX, USA).

### 2.2. Animals, Treatment, and Sample Collection

Male Sprague-Dawley (SD) rats (7 weeks old) were supplied by SPF Biotechnology Co. Ltd. (Beijing, China). All experimental procedures were approved by the Animal Ethics Committee of Beijing University of Chinese Medicine (No. 2015BZHYLL0201) and followed the Regulations for Laboratory Animal Management. SD rats were maintained on a 12 h light/dark cycle at 22 ± 2°C with ad libitum access to a standard chow diet (n = 10) or a HFD (34% fat, 19% protein, and 47% carbohydrate by energy composition) for 8 weeks to induce NAFLD. Animals were randomly divided into the following experimental groups (each n = 10): control group, fed a standard chow diet and oral saline (10 mL/kg/day); HFD model group, fed a HFD and oral saline (10 mL/kg/day); GLU group, fed a HFD and oral GLU (1.5 g/kg/day); GGQLL group, fed a HFD and low dose GGQLD (1.26 g/kg/day); GGQLS group, fed a HFD and solid dose GGQLD (2.52 g/kg/day); and GGQLH group, fed a HFD and high dose GGQLD (5.04 g/kg/day). The GGQLD granules and GLU were dissolved in 100 mL of distilled water and kept at 2–8°C until use. Feedings occurred daily during the 8-week experimental period. Fresh stool samples were collected at the end of the eight-week treatment and stored at −80°C until further analysis.

### 2.3. Sequencing Analysis of Microbial Diversity

We followed the methods of Shin et al. (2017) [35]. DNA was extracted from the fecal samples by standard, published protocols. Three relatively conserved variable regions (V1, V2, and V3) of 16S rRNA were performed using a C1000 Touch thermal cycler (Bio-Rad, Hercules, CA, USA).

The obtained sequence data were sorted by their unique barcodes in a demultiplexing step, and low-quality reads (average quality score <25 or read length <300 bp) were not considered for analysis. Operational taxonomic units (OTUs) were defined at a cutoff of 97%. Sequences were assigned to OTUs (Greengenes Database: http://greengenes.lbl.gov) followed by the selection of the representative sequence using the Quantitative Insights into Microbial Ecology (QIIME) software package. Phylogenetic Investigation of Communities by Reconstruction of Unobserved States (PICRUSt) was performed to identify functional genes in the sampled microbial community on the basis of the data in the Kyoto Encyclopedia of Genes and Genomes (KEGG) pathway database.

To identify taxa with differential relative abundance in each experimental group, the linear discriminant analysis (LDA) effect size (LEfSe) method was used. For this analysis, a web-based program was employed with the following conditions: the alpha value of the factorial Kruskal-Wallis test among classes was set to <0.05 and the threshold of the logarithmic LDA score for discriminative features was set to >2.0.

## 3. Results

**Figure 1:**
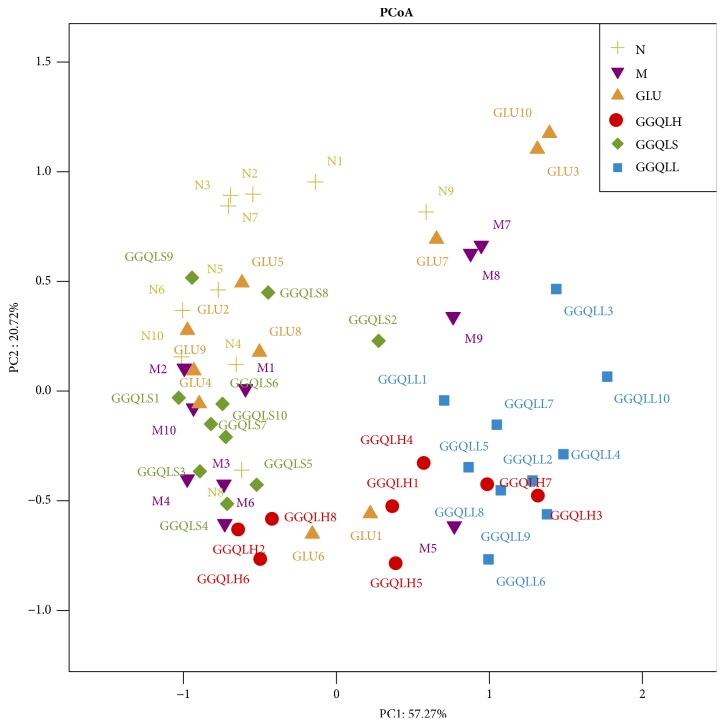
Principal component analysis (PCoA) score plot calculated from the operational taxonomic unit (OUT) levels with QIIME software and unweighted UniFrac analysis.

To profile the effect of GGQLD on gut microbial structure and composition in NAFLD rats, stool samples were sequenced for the 16S rRNA gene. According to our principal component analysis (PCoA), which divided the microbiome according to species composition, the gut composition of the HFD group was easily distinguished from the control, GGQLH, and GGQLL groups (Figure 1). Furthermore, the distance between the HFD and GGQLL groups was greater than that between the HFD group and both the GGQLH and GGQLS groups.

**Figure 2:**
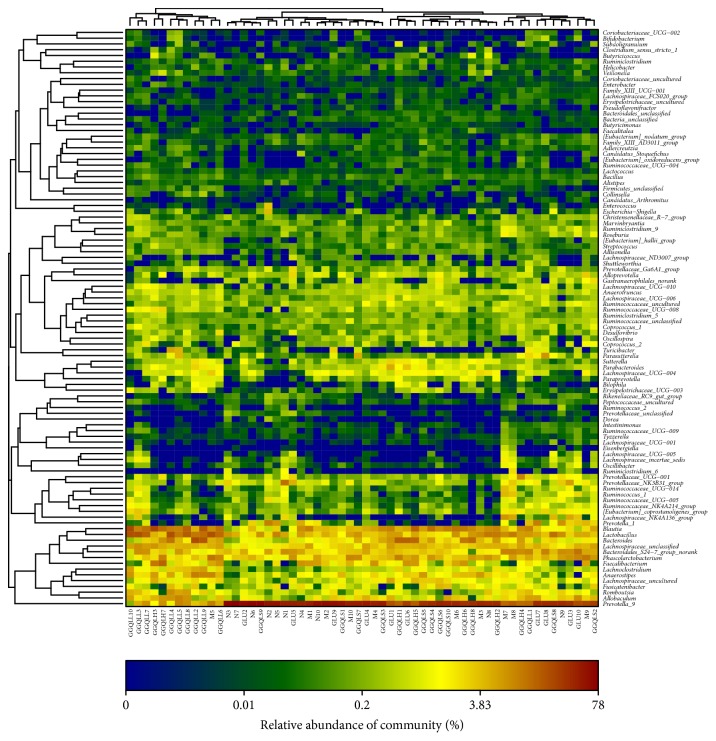
Heatmap and clustering of individual gut microbiota for 150 operational taxonomic units (OTUs).

**Figure 3:**
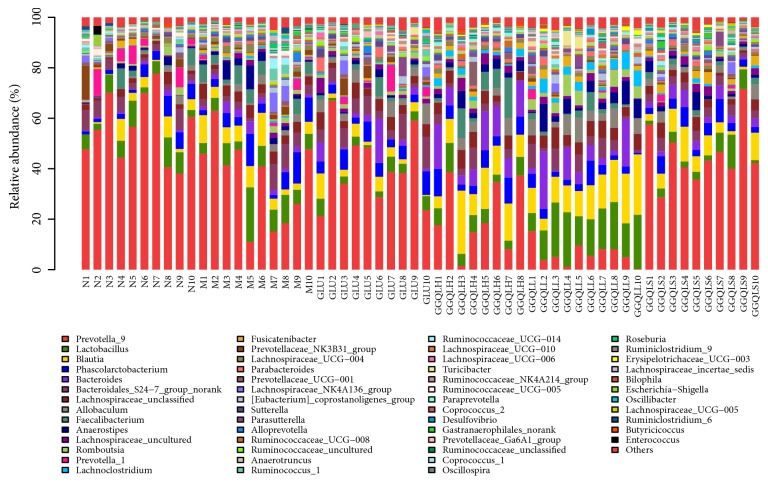
Relative abundance of various microbial families in each gut microbiota sample.

The microbial composition for each group was further analyzed at the phylum and genus levels. A heatmap analysis of 150 OTUs showed that the gut microbiome composition of the GGQLL group was closely related to that of the control group, both of which were different from the HFD group (Figure 2). Bacteroidetes was the dominant class in the control, GLU, and GGQLS groups, contributing to 69.6%, 57.7%, and 56.2% of the total fecal microbial population, respectively. In contrast, Firmicutes was the dominant class in the HFD, GGQLH, and GGQLL groups, contributing to 50.9%, 57.1%, and 74.7% of the total fecal microbial population, respectively. Like phylum, microbial family also varied greatly among the experimental groups. The dominant family was Prevotella_9 in Bacteroidetes contributing to 56.1%, 35.7%, 40.8%, 21.4%, and 45.6% of the total fecal microbial population in the control, HFD, GLU, GGQLH, and GGQLS groups (Figure 3). The only exception was the GGQLL group, whose dominant family was Lactobacillus contributing to 15.8%.

**Figure 4:**
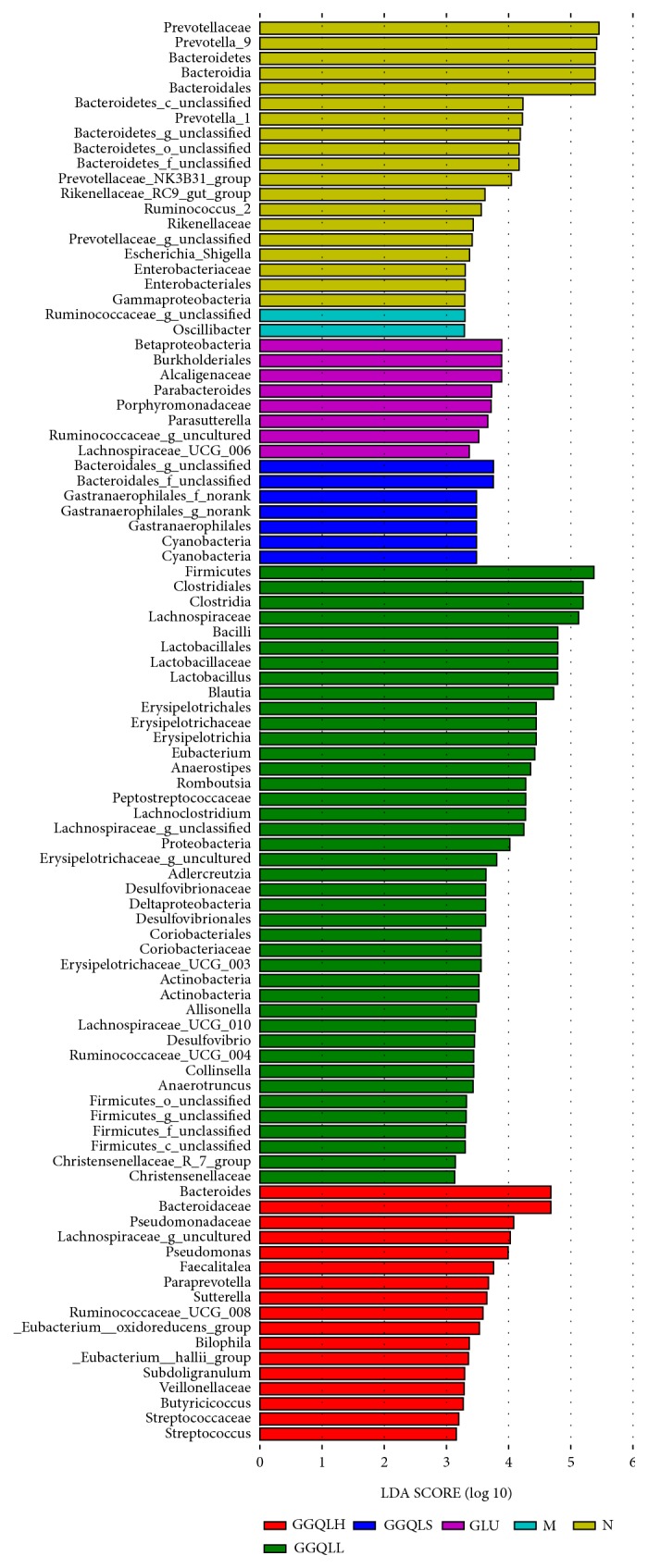
Cladogram showing the differentially abundant gut microbial taxa between experimental groups.

Next, the LEfSe method was used to determine the differentially abundant microbial taxa between the experimental groups. The cladogram from the LEfSe results revealed that, compared with the other groups, two taxa were increased in the HFD group (Figure 4). In the control, GLU, GGQLS, GGQLL, and GGQLH groups, a total of 19, 8, 7, 42, and 17 taxa were increased, respectively. Collectively, these results indicate that the gut microbial composition was differentially modulated in GGQLL rats in response to being fed HFD.

## 4. Discussion

It is important to note that there are numbers of studies that have investigated microbiota composition in patients with simple steatosis or NASH, However, the results are controversial. Indeed, changes in the* Bacteroidetes*,* Lachnospiraceae, *and* Ruminococcaceae *families show completely opposite tendencies in different studies [21, 36, 37]. Therefore, further evaluation of these changes in the gut microbiome during NAFLD is essential. Nowadays, the 16S rRNA method has been widely used for gut microbiome. Shin et al. set for us a good example of CHM research for treating NAFLD via gut microflora [35].

The mechanisms by which gut bacteria affect the symptoms of NAFLD are still largely unknown. However, some complicated processes have been indicated to be involved. Microbial populations of NASH patients have been proven to have a strong ability to produce ethanol [38] and some components of the gut microbiota can transform choline to trimethylamine, both of which can result in liver injury leading to hepatic steatosis and steatohepatitis [39, 40]. Furthermore, dysbiosis of gut microflora has been suggested to be related to changes in the level of serum metabolites, such as branched-chain amino acids (BCAAs) and aromatic amino acids (AAAs). Furthermore, BCAAs are increased in individuals with IR, which is regarded as the main risk factor of NASH [41].

In the present study, we utilized a HFD to induce NAFLD in rats. HFD feeding is extensively used in rodents to model obesity, steatosis, and insulin resistance. In fact, these models are considered more relevant to human metabolic diseases than other models of gene inactivation. By exploiting the close connection between intestinal permeability and the gastrointestinal microbiome, which represent the biological barrier in the gut, we investigated the relative abundance of different taxa in this HFD model. In our analysis, we observed HFD-dependent differences at the phylum, class, and genus levels that resulted in dysbiosis. These changes were characterized by an increase in the Firmicutes/Bacteroidetes ratio and a dramatic increase in the Oscillibacter genus compared to the control group.

Interestingly, studying the gut microbiome not only highlights disease-related changes, but the composition of the flora can also reflect the effectiveness of treatment. Although The proposed NAFLD is typically treated with lifestyle modifications and/or pharmaceutical intervention [7, 19, 20], these are associated with issues that limit their effectiveness. Furthermore, probiotic treatments to modulate the gut microbiome specifically still require further investigation before they can be recommended as a treatment for NAFLD [22, 23]. In the present study, we evaluated the effectiveness of GGQLD, a CHM that is commonly prescribed to treat NAFLD/NASH. We focused on the ability of this herbal formula to modulate the gut microbiome. In our analysis, GGQLD improved the HFD-induced changes in the intestinal flora, especially in the GGQLL group, which had increased levels of Firmicutes, Clostridia, Lactobacillus, bacilli, and Erysipelotrichales. These data are supported by other studies reporting similar results [42].

In a previous study, we found that GGQLD treatment reduced serum sIgA levels in this HFD-induced NASH rat model in addition to increasing zonula occludens-1 (ZO-1) expression and reducing prostaglandin E2 (PGE2) expression in the gut [29]. ZO-1 is a tight junction protein that is closely related to gut function, while PGE2 is a bioactive lipid that mediates inflammation. These data indicate that GGQLD may enhance the barrier function of the gut, while also suppressing inflammation. Furthermore, we also found that GGQLD regulated lipid aggregation and improved hepatic peroxisome proliferator-activated receptor-gamma (PPAR-*γ*) and insulin-resistance (IR) expression in vivo and in vitro [27], further highlighting the anti-inflammatory function of this CHM. Indeed, these changes appear to be modulated via the Sirt1 pathway. Sirt1, a regulator of PPAR-*γ* coactivator-1 alpha (PGC-1*α*), induces the transcription of metabolically relevant genes involved in the oxidation of mitochondrial fatty acids [43]. This cascade has a negative regulatory effect on inflammatory processes. Although some previous studies suggest that SIRT1 might prevent intestinal inflammation by regulating the gut microbiota [44], a theory that is supported by the present study, the interaction between the Sirt1 pathway and gut flora requires further investigation. Besides, some experiments suggest a potentially causal role of the gut microbiome and gut-microbiome-derived metabolites in the development of NAFLD such as phenylacetic acid, a novel microbial metabolite involved in AAA metabolism, which was identified as a key gut-microbiome-derived metabolite associated with hepatic steatosis. Therefore, we will explore the interaction between the gut microbiome, the liver, and metabolism under the effect of GGQLD through metabonomics.

In conclusion, we have shown that GGQLD treatment is effective in treating NAFLD/NASH via modulation of the gut microbiome. Indeed, the disease-related changes in abundance and distribution of different taxa in the gut microbiome that were observed in the HFD-induced NAFLD/NASH model were largely remedied following treatment with GGQLD, particularly GGQLL, which returned the microbiome to a similar composition as that of the untreated controls. To our knowledge, this is the first time the effects of GGQLD have been assessed with regard to gut microflora in NAFLD/NASH. Taken together, our data highlight the efficacy of GGQLD in treating NAFLD and support its clinical use as a treatment for NAFLD/NASH patients.

## Data Availability

The data used to support the findings of this study are available from the corresponding author upon request.
